# Headache attributed to airplane travel: diagnosis, pathophysiology, and treatment – a systematic review

**DOI:** 10.1186/s10194-017-0788-0

**Published:** 2017-08-16

**Authors:** Sebastian Bao Dinh Bui, Parisa Gazerani

**Affiliations:** SMI®, Department of Health Science and Technology, Faculty of Medicine, Aalborg University, Aalborg, Denmark

**Keywords:** Headache attributed to airplane travel, Airplane headache, Diagnosis, Pathophysiology, Treatment, Sinus barotrauma, NSAIDs, Triptans

## Abstract

**Background:**

Headache attributed to airplane travel, also named "airplane headache" (AH) is a headache that occurs during take-off and landing. Today, there are still uncertainties about the pathophysiology and treatment of AH. This systematic review was performed to facilitate identification of the existing literature on AH in order to discuss the current evidence and areas that remain to be investigated in AH.

**Methods:**

The systematic literature search was performed in 3 relevant medical databases; PubMed, Scopus, and Embase. The search yielded 220 papers and the papers were sorted based on inclusion and exclusion criteria established for this study.

**Results:**

This systematic review included 39 papers. Main findings revealed that AH attacks are clinically stereotyped and appear mostly during landing phases. The headache presents as a severe painful headache that often disappears within 30 min. The pain is unilateral and localized in the fronto-orbital region. Sinus barotrauma has been considered as the main cause of AH. Nonsteroidal anti-inflammatory drugs and triptans have been taken by passengers with AH, to relieve the headache.

**Conclusions:**

Based on this systematic review, further studies seem required to investigate underlying mechanisms in AH and also to investigate the biological effects of nonsteroidal anti-inflammatory drugs and triptans for alleviating of AH. These studies would advance our understanding of AH pathogenesis and potential use of treatments that are not yet established.

## Introduction

Headache attributed to airplane travel, also named "airplane headache" (AH) occurs in a population of passengers during airplane travels. The headache appears as an intense short lasting pain at landing and it is often located in the fronto-orbital region [1–7]. Despite its occurrence rate and high impact, only limited is known about AH, and this type of headache has only been defined and included in the headache classification since 2013 by International Headache Society (IHS), which provides headache classifications and maintains related updates [1]. The first case of AH was described in 2004, and since then, number of publications on AH has been added into the literature [2–25]. Previous reports, before inclusion of AH in classification, could be based on diversity in diagnosis, which makes it difficult to determine whether reported patients suffered from AH or other conditions [1, 6]. Despite the fact that some points are known based on these publications, there is still uncertainty around influence of ethnicity, gender or age on incidence or prevalence of AH. A male dominancy has been reported for AH [4, 6, 26]. Current knowledge about pathophysiology and treatment of AH is limited that calls for further investigation on both epidemiological aspects of AH, pathophysiology and treatment options.

There is also a diverse range of hypotheses about the pathophysiological causes of AH. Previous studies have suggested vasodilation in the cerebral arteries or sinus barotrauma as a result of cabin pressure change in the airplane [2, 3, 6, 11, 12, 26]. These proposed mechanisms require further investigation to prove or falsify suggested theories. Besides, no specific treatment plan has been developed for AH, although several medications have shown beneficial effects, e.g. triptans [11]. Considering challenges and limitation of AH studies under real-time conditions, it might be an option to study this headache under controlled experimental conditions. This approach has also been used in studying other types of headaches [25]. An experimental model of AH has been developed recently [25] that can help in further understanding of potential mechanisms underlying AH, or to examine AH under different circumstances, and identification of potential biological biomarkers. This model [25] can also serve for testing treatment options for AH.

To provide a better overview of existing literature on diagnosis, pathophysiology, and treatment of AH, this systematic review was performed. It was proposed that outcome of this review would highlight existing evidences, missing information, and stimulate further research in AH. Understanding AH pathogenesis and efficient targeting would ultimately help millions of passengers who suffer from this condition.

## Methods

### Literature search

Both authors (SBDB and PG) contributed in performing the systematic literature search in PubMed, Scopus, and Embase by using the terms “airplane headache” and “aeroplane headache” (airplane OR aeroplane AND headache). PubMed was searched on 2nd March 2017 (January 2004 to March 2017), Scopus was searched on 3rd March 2017 (January 2004 to March 2017) and Embase was searched on 5th March 2017 (January 2004 to March 2017).

### Selection criteria and data extraction

Due to limited information available about AH, all types of studies and levels of evidence about AH were considered eligible for inclusion, including i.e. case series, case reports, conference abstracts, and all types of publications providing knowledge on diagnosis, pathophysiology, or treatment of AH. All papers were exported and duplicates were excluded through the Excel 2010 (Microsoft Corp., Seattle, WA, USA). Titles that appeared relevant were assessed for inclusion. Papers were excluded if they were not available in English. All data from the included papers were reviewed and tabulated according to authors of study, year, study type, demographic data on the patients, and main outcomes.

## Results

### Literature search

**Fig. 1:**
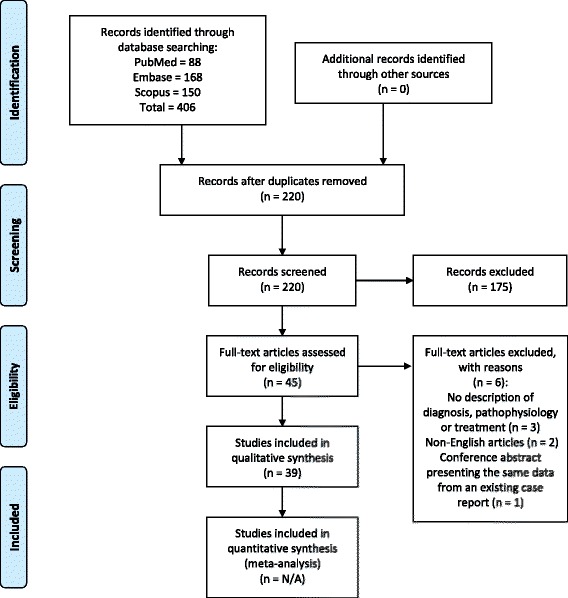
PRISMA flowchart for the selection of studies

The flowchart for selection of papers is shown in Fig. 1. The search strategy identified 220 papers. The authors excluded 175 papers due to lack of relevance to AH. Furthermore, 6 additional papers were excluded as those did not contain relevant description of AH, were not written in English, and reported the same data from an existing case report on AH. The authors, therefore, included 39 papers for the final review, which provided sufficient information on diagnosis, pathophysiology and/or treatment of AH (see Table 1).

**Table 1:** Demographic characteristics and main outcomes of the current literature regarding headache attributed to airplane travel

Year, Author (reference)	Number of AH-patients	Gender (number of M/F)	Mean diagnosis age (mean ± SD) years	Publication type	Main outcomes
2004, Atkinson et al. [8]	1	1 M	28	Case report	• Patient developed a severe headache during ascent and descent. The pain was described as jabbing in the unilateral fronto-orbital region and lasted within 20 min. • Patient had no relevant medical history, used no medication and had no symptoms of sinus disease. • Neurological examinations were performed, CT and MRI, with normal findings.
2006, Berilgen et al. [7]	6	6 M	37.3 ± 2.9	Case reports	• All 6 patients experienced a severe headache when the airplane was taking off or landing. The pain was described as jabbing and stabbing in the orbital and/or supraorbital region. The headaches resolved within 15–20 min. • Three patients reported a history of migraine without aura, retinal migraine and exercise headache. • The authors suggested sinus barotrauma as the main cause of the headache. The pressure changes during the ascent and descent would potentially activate the trigeminovascular system and thereby induce the headache attack. • Examinations of neurological, ENT, blood analyses, MRI and paranasal sinus tomography showed normal findings.
2007, Evans et al. [9]	4	2 M/2 F	26.8 ± 3.9	Case reports	• All patients developed severe headache during the descent that lasted about 15–30 min. The pain intensity was rated as 10/10 and located in parietal and ethmoid regions. • Two patients experienced a second mild/moderate headache after the AH. • Ibuprofen was used by two patients; one patient experienced a mild reduction in pain and one did not experience any relieving effect. • The authors stated that sinus barotraume might be an explanation for the cause of the AH. • Examinations of CT, MRI and ENT showed normal findings.
2007, Mainardi et al. [5]	1	1 M	23	Case report	• The patient developed a severe pulsating headache after the take-off in the retro-orbital and frontal region. The headache lasted about 10–15 min. • Patient had no relevant medical history. • Authors believed that sinus barotrauma might not explain the cause of the headache as this patient developed the headache after the take-off. • Neurological and neuroradiological examinations (MRI and MRA) showed normal findings.
2008, Marchioretto et al. [15]	1	1 M	29	Letter to the editor	• The patient developed a highly sharp intense headache pain during the landing in the periorbital region. The pain resolved after 5 min. • Sodium naproxen 550 mg, one tablet, was taken by the patient after the first incidence of headache. He did not experience any headache after the use of the medication. • Neuroradiological examinations (MRI and MRA) showed normal findings.
2008, Coutinho et al. [17]	1	1 M	57	Case report	• The patient always developed an intense headache pain in the left frontal region during the landing. The headache often lasted between 5 and 10 min. • There was no relevant medical history and no history of sinus pathology. • Neurological examination, ENT and MRI, showed normal findings. • The authors excluded sinus barotrauma as a possible explanation of the headache due to the normal findings of the MRI.
2008, Potasman et al. [4]	52	18 M/34 F	33.3 ± 13.8	Journal article	• The travelers developed headache during the ascent and descent. The pain was often described as a unilateral or bilateral pressuring sensation with a pain intensity of 6 (on a scale of 1–10). • The headache lasted 4.0 h after take-off and 5.7 h after landing. • In the headache group, 23 passengers were diagnosed with migraine, 3 passengers reported sinusitis as the cause of the headache and 3 passengers reported hypertension as the cause. • Medications such as paracetamol, dipyrone and triptans, were taken by 25 travelers. The effects were not reported.
2008, Kim et al. [27]	1	1 M	38	Case report	• The patient developed a severe headache during the landing. The pain was described as jabbing and located in the supraorbital area. The headache lasted about 30 min. • Neurological examinations, x-ray of paranasal sinuses and cranial CT showed overall normal findings, except for mucosal thickening of the right ethmoid sinus. • Authors stated that the results of the thickening ethmoid sinus mucosa might be an incidental finding.
2010, Baldacci et al. [13]	1	1 F	20	Case report	• The patient experienced a sharp pain in the right retro-orbital and frontal regions during the take-offs. The pain sensation was severe with the rating 10/10 and the headache disappeared within 10 min. • There was no relevant medical history; but, every headache attack was preceded with a characteristic sensory aura. She always experienced a sensation of paraesthesias starting from the left thumb accompanied by spread to her hand and to the perioral region. These symptoms lasted 5–10 min before the start of the headache attack. • Neurological examinations, ENT, EEG, CT and MRA showed normal findings. • The authors stated that sinus barotrauma might explain the mechanism for the headache and suggest that the reported aura might be related to the pressure changes in the cabin.
2010, Domitrz [10]	1	1 M	29	Case report	• The patient developed a sudden jabbing headache during the take-off and landing – but only when he was travelling with airplane and not with jumbo jet. The pain was located in the left frontal region with radiation into the left eye. The intensity of the pain was severe and the patient could not move until the headache disappeared. • Medical history was not relevant. Neurological-, ophtolaryngological-, and ophthalmological examinations and MRA showed normal findings. • The author stated that sinus barotrauma might be a possible cause of the headache.
2010, Ipekdal et al. [28]	2	1 M/ 1 F	12 ± 1	Conference abstract	• Two children suffered from severe headaches during the landing phase. • Blood sample analyses, cerebral MRI and MRA showed normal findings. However, paranasal sinus tomography revealed pansinusitis in the male patient and bilateral maxillary sinusitis in the female patient. • Both patients were given antibiotic and anti-inflammatory medications and experienced afterwards headache-free airplane travels.
2010, Ipekdal et al. [16]	3	1 M/ 2 F	13 ± 0.8	Case reports	• Three children developed severe headaches during the ascent and descent. The pain was located in the unilateral periorbital and orbito-frontal region and lasted between 10 and 25 min. • One patient had a history of allergy rhinitis and allergy to pollens. • Paranasal sinus tomography and MRI showed nasal mucosal wall thickening and inflammation in all 3 patients. ENT showed adenoidal and tonsillar hypertrophy in one patient. • The patient with adenoidal and tonsillar hypertrophy underwent adenotonsillectomy surgery and experienced headache-free airplane travels after recovery. • The 2 other patients with inflamed nasal mucosal walls were given antihistamine and anti-inflammatory medications and were completely headache-free after treatment. • The authors stated that their effective treatment with antihistamine and anti-inflammatory medications support the theory that sinus barotrauma plays a central role in the mechanism of AH.
2010, Pfund et al. [19]	1	1 F	27	Case report	• The patient developed bilateral headache located in her ear, cheek, forehead, and on the top of the head. The pain was described as stabbing, jabbing and rated as 10/10. • The severe headache pain disappeared after landing, but was followed by a mild headache that lasted two weeks after the flight travel. • MRI and paranasal sinus computed tomography and revealed bilateral inflammation in the sphenoid, maxillary and ethmoid sinuses. Furthermore, the patient had increased number of eosinophilic cells and swelled nasal mucosa. These findings supported the diagnosis of chronic non-allergic rhinosinusitis. • Patient was given antihistamine and experienced almost painless airplane travels. • The authors supported the sinus barotrauma as a central role in the mechanism of AH.
2011, Berilgen et al. [26]	18	16 M/ 2 F	34.25 ± 7.7	Case reports	• All patients developed a severe headache during the landing phase. The pain was located in the frontal and orbital region and described as jabbing and stabbing with a duration of 15–30 min. • All ENT and neurological examinations showed normal findings. • The authors supported the hypothesis of sinus barotrauma as a possible mechanism of AH.
2011, Mainardi et al. [29]	N/A	N/A	N/A	Letter to the editor	• The authors stated that the findings from the paper by Pfund et al. [19] differ from the stereotypical AH-attacks: 1) The AH-attack does not exceed 30 min, 2) the pain is not strictly bilateral and 3) neuroradiological examinations do not show signs of sinus inflammation. • They suggested that “pure” AH should be considered as a separated headache from the flight-related headache in patients with organic condition.
2011, Ipekdal et al. [11]	5	1 M/ 4 F	29.6 ± 1.9	Case reports	• All the patients developed a severe headache during the ascending or the descending phase. The pain was located in the unilateral fronto-orbital region and lasted between 15 and 25 min. • Triptans were prescribed to all patients and they were instructed to take a triptan (either naratriptan, zolmitriptan, eletriptan or sumatriptan) 30 min before the start of the airplane travel. There was a follow-up period of 2–4 years and all patients were completely headache-free in all their airplane travels. • The authors indicated that sinus barotrauma might play a role in the mechanism of AH. They also hypothesized that the use of triptans may cause vasospasm in cerebral arteries and thereby prevent the AH-attack.
2011, Mainardi et al. [30]	N/A	N/A	N/A	Letter to the editor	• The authors suggested that AH should be included in the forthcoming revision of the “International Classification of Headache Disorders” in the section of “Headache attributed to disorder of homoeostasis”. • They stated that the criteria set by Berilgen et al. [26] are broadened. The criteria of AH should be restrictive and clearly defined in order to properly diagnose the headache.
2011, Mainardi et al. [31]	63	41 M/ 22 F	29.5	Conference abstract	• The headache occurred mainly at landing and in the fronto-orbital region. Pain intensity was severe and lasted about 20 min. • Prophylactic use of NSAIDs prevented the AH-attacks. • The authors stated that their findings confirm the stereotypical features of AH and that the headache should be recognized by The International Headache Society.
2011, Kararizou et al. [12]	1	1 F	27	Case report	• The patient experienced an intense headache in the frontotemporal and retro-orbital area of the skull. The pain was described as jabbing and lasted about 15–20 min. • She had a history of tension type headache. ENT, CT, MRI and MRA showed normal findings. A test for anxiety and depression was performed with a score of 14 on the Hamilton anxiety scale (mild anxiety ≥14) and 12 on the Hamilton depression scale (mild depression >7). • Paracetamol and ibuprofen were taken by the patient, but there was no relieving effect. • The authors stated that psychiatric disorders should be investigated for future studies in order to establish a possible signification to AH. This might clarify the mechanism of AH and specify effective therapeutic strategies.
2012, Mohamad [24]	N/A	N/A	N/A	Letter to the editor	• The author ascertained that the clinical features of AH are similar with the symptoms of sinus barotrauma. According to author, the pressure changes during the landing phase might be a key player in the pathophysiology of AH. • According to author, sinus barotrauma could be prevented by surgical or medical intervention.
2012, Purdy [14]	N/A	N/A	N/A	Editorial	• The author postulated that sinus barotrauma might be the main mechanism in the pathophysiology of AH. • According to the author, the increasing number of case reports on AH is important for the further research for this headache that will allow future studies to examine the mechanism and thereby develop specific therapeutic strategies.
2012, Shevel [32]	N/A	N/A	N/A	Letter to the editor	• The author stated that it does not seem plausible that the changes in the barometric pressure should trigger the AH-attack during the ascending or descending phases. The pressure in the sinus is low during the whole flight travel and will not be affected by the increasing ambient pressure during the descending phase. • The pressure in the cabin and the intra-sinus pressure is the lowest during the whole flight travel. The pain is expected to occur during this phase, which is not the case.
2012, Mainardi et al. [6]	75	29 M/ 46 F	36.5 ± 10.2	Journal article	• The majority of the patients developed a headache during the landing. The headache lasted less than 30 min. In most of the cases, the pain was described as jabbing, stabbing and located in the fronto-orbital region. The intensity of pain was severe and rated as 8.8/10. • A subgroup, consisting 29 AH-patients, took medications. Only 11 of the patients were completely headache-free when they used ibuprofen, naproxen, aspirin and nasal decongestant. • A minority of the patients used non-pharmacological methods such as Valsalva manoeuvre, pressure on pain site chewing. These methods showed an unremarkable efficacy. • The authors did not think that the change in pressure is the only trigger for the AH-attack. Other factors such as environmental factors (aircraft speed, angle of ascent/descent, maximum altitude) and anatomic factors (acquired or congenital abnormalities of sinus outlet might contribute to the development of AH.
2013, Mainardi et al. [33]	11	5 M/6 F	37	Conference abstract	• All patients suffered from AH and experienced a similar headache when they were descending a mountain by car. Both headaches were described as a severe jabbing pain in the unilateral fronto-temporal region and lasted about 20 min. • General and neurological examination, brain MRI, MRA, and cranial CT-scan for sinuses showed normal findings. • The authors suggested that both headaches might share a possible common pathophysiology mechanism as both headaches are trigged by changes in the pressure.
2013, Mainardi et al. [34]	9	4 M/5 F	37 ± 12	Conference abstract	• All AH-patients experienced a similar headache when they were scuba diving. The headache pain started shortly after the ascent. • MRI, MRA and cranial CT-scan for sinuses showed normal findings. • The authors suggested that AH and headache attributed to scuba diving might share a common pathophysiology mechanism as both headaches are trigged by external pressure.
2013, Mainardi et al. [3]	N/A	N/A	N/A	Journal article	• The authors stated that AH might share a common physiological mechanism with the situations when you are descending from a mountain in car or diving as all these conditions are trigged the changes in the pressure. • Beside the pressure changes, other factors such as environmental factors (aircraft speed, angle of ascent/descent, maximum altitude) and anatomic factors (acquired or congenital abnormalities of sinus outlet might contribute to the development of AH. • According to the authors, AH should be considered as a formal headache by “International Headache Society”.
2013, Cherian et al. [35]	2	2 M	33 ± 1	Case reports	• Both patients developed headache during the descent. The pain was described as jabbing, stabbing and located in the unilateral supraorbital region. The intensity of pain was severe and rated as 9.5/10. • MRI and CT showed normal findings. Only one patient had a history of migraine without aura. • The patients took oxymetazoline nasal drops in combination with naproxen sodium which completely prevented the AH-attacks.
2013, Nagatani [21]	2	1 M/1 F	37.5 ± 11.5	Case reports	• Both patients developed a severe headache during the descent. The headache lasted 30–40 min and the pain was located in the fronto-orbital region. • Medical history was not relevant for the female patient, but male patient had had a past history of episodic tension-type headache. Intracranial and paranasal CT examinations showed normal findings. • For the male patient, the headache only occurred when he had mental stress or was suffering from a lack of sleep. • NSAIDs were taken by both patients, but did not show any relieving effect. • The authors pointed out that the prevalence of AH might be underestimated as many passengers, suffering from AH, do not seek a doctor.
2015, Rogers et al. [18]	1	1 F	11	Case report	• The patient experienced a severe headache during the ascent. The pain was described as sharp, stabbing, throbbing and located in the unilateral and frontal region. Every headache episode was associated with dizziness, but there were no additional accompanying symptoms. • Medical history was significant for episodic migraine and adenotonsillectomy only. • Blood test, MRI and EEG showed normal findings. • The authors stated that their data, in conjunction with the reignition of AH by the International Headache Society, contribute to the increasing evidence on AH.
2015, Mainardi et al. [36]	130	76 M/54 F	N/A	Conference abstract	• The majority of the patients developed AH during the landing phase. The pain was located in the frontal-orbital region and lasted less than 30 min. • The AH-attacks occurred more than 50% of the flight travels in 35 patients and 23 patients experienced AH in every flight travel. • Prophylactic use of NSAIDs showed a relieving or preventing effect in more than 50% of the cases. • The authors stated that these findings confirm the stereotypical clinical features of an AH-attack.
2015, Mainardi et al. [37]	140	83 M/ 87 F	N/A	Conference abstract	• The data from this publication are similar to the conference abstract by Mainardi et al. [36].
2016, Hiraga et al. [23]	N/A	N/A	N/A	Case report	• A 74-year old woman experienced a severe headache during the descent. The headache met the criteria of “International Classification of Headache Disorders 3 beta version” [1] for AH. • The headache pain continued for 48 h after landing. • MRA showed segmental vasoconstriction of brain vessels allowing the diagnosis reversible cerebral vasoconstriction syndrome (RCVS). • The authors suggested that RCVS may play a role in the pathophysiology of AH.
2016, Mainardi et al. [22]	N/A	N/A	N/A	Editorial	• The authors disagreed with the findings by Hiraga et al. [23] and ascertain that the clinical features of reversible cerebral vasoconstriction syndrome (RCVS) and AH are not comparable. By instance, the duration of an AH-attack does not exceed 30 min. The authors emphasized that patients who are suffering a second phase headache after an AH-attack should be investigated carefully in order to rule out secondary pathology, including RCVS.
2016, Hiraga et al. [38]	N/A	N/A	N/A	Editorial	• The authors agreed with Mainardi et al. [22] that AH and reversible cerebral vasoconstriction syndrome (RCVS) are two separated types of headache. • However, they suggested that RCVS might be a potential cause of AH in cases of second headaches arising after the resolution of the triggering factors such as the airplane descent. As a subgroup of AH sufferers do experience a second phase headache after the AH-attack, the authors believed that RCVS might be an overlooked condition and the physicians should be aware that airplane descent might be a trigger of RCVS.
2016, Mainardi et al. [20]	1	1 M	36	Case report	• The patient experienced a headache when she was descending from high altitude by car, called Mountain Descending Headache (MDH). The clinical features of the headache were identical to the stereotypical features of AH. • The authors suggested that MDH and AH might share a common pathophysiology mechanism as both headaches are trigged by the imbalance between the pressure in the sinuses and the changing environmental pressure.
2016, Zubero et al. [39]	1	1 F	34	Case report	• The patient developed a severe predominant right hemicranial headache with rhinorrhea and tearing during take-off and landing. • Medical history was not relevant. Neurological examinations showed normal findings.
2016, Bui et al. [2]	21	9 M/12 F	39 ± 14	Journal article	• All patients fulfilled the AH-criteria set by “International Classification of Headache Disorders 3 beta version” [1]. • Seven patients suffered from migraine and 13 patients suffered from High Altitude Headache. • The statistical analysis indicated that High Altitude Headache, but not migraine, might be a risk factor for AH. • Five patients took medication, i.e. paracetamol and triptans. These medications were effective to relieve and prevent the headache pain. • The authors supported the established hypothesis that changes in the pressure might play a key role in the mechanism of AH.
2017, Nath et al. [40]	2	2 M	45.5 ± 3.5	Case reports	• The patients experienced a bilateral occipital and parieto-occipital severe sharp shooting stabbing and piercing headache during the landing. The headache was associated with severe dizziness and lasted 40–50 min after landing. Both patients rated the intensity of pain as 8–9/10. • ENT, CT and MRI showed normal findings. Both patients had no history of tension headache, migraine or cluster headache. • The authors ascertained that the duration of the headache and the pain localization in the occipital and parieto-occipital area are not in accordance with the AH-criteria set by “International Classification of Headache Disorders 3 beta version” [1].
2017, Bui et al. [25]	7	1 M/ 6 F	29.7 ± 9.6	Journal article	• All patients fulfilled the AH-criteria set by “International Classification of Headache Disorders 3 beta version” [1]. • A pressure chamber was used as an experimental model to induce simulated AH in the patients. The clinical features of the headache were in accordance with an AH-attack in a real-time flight travel. • Saliva samples, saturation pulse oxygen, blood pressure and thermo imagining were collected and measured before, during and after their simulated flight in the pressure chamber. • The data showed that the values for saturation pulse oxygen, prostaglandin E_2_ and cortisol significant different in AH-patients in comparison with the healthy participants.

*AH* Airplane headache also called headache attributed to airplane travel. *CT* computerized tomography. *MRI* brain magnetic resonance imagining. *MRA* magnetic resonance angiography. *ENT* ear-nose-throat, *EEG* Electroencephalography, *NSAIDs* Non Steroidal Anti-Inflammatory Drugs

### AH diagnosis

The clinical diagnostic data are presented as cumulative from all included papers (Table 1) [2–40]. In this cumulative synthesis, 275 patients are represented [2, 4–13, 15–19, 21, 25–28, 31, 33–37, 39, 40]; 127 females (46%) and 148 males (54%). The median age at diagnosis was 28.7 ± 4.8 (mean ± SD) years (*n* = 275/275) and the median age of onset of the first AH-attack was 26.4 ± 3.8 (mean ± SD) years (reported by 163/275 patients).

#### Duration, severity and frequency of AH

Collectively, based on available evidences, landing appears to be the phase of the flight during which most of patients experience AH-attack with a duration within 30 min. During the AH-attack, the pain is described as severe with a rating of 8–10 on a scale from 0 (no pain) to 10 (worst possible pain), (*n* = 229/275). In few cases, patients have had experienced a second mild phase headache after the AH-attack that resolved within 4–24 h (*n* = 2/275). The frequencies of the AH-attacks were reported by some patients: 42 patients experienced AH in every flight travel, while AH occurred in more than 50% of the flight travels in 39 patients.

#### Symptoms characteristics of AH

The AH-attack is often experienced as jabbing, stabbing and/or pulsating in the unilateral fronto-orbital region and is not associated with accompanying symptoms in most of the AH-attacks. However, few patients did experience these accompanying symptoms; dizziness (*n* = 2/275), sensation of paraesthesias starting from the left thumb accompanied by spread to the hand and perioral region (*n* = 1/275).

#### Medical history

The majority of the AH-patients did not have any relevant medical history. However, some patients had a history of migraine (*n* = 54/275), tension type headache (*n* = 22/275), allergy (*n* = 25/275), and chronic non-allergic rhinosinusitis (*n* = 1/275). A minority of patients reported that they also suffered from High Altitude Headache (*n* = 13/275, Mountain Descending Headache (*n* = 11/275), and Scuba Diving Headache (*n* = 9/275).

#### Neurological examinations

Neurological examinations, such as brain magnetic resonance imagining (MRI), magnetic resonance angiography (MRA), ear-nose-throat (ENT) and/or computerized tomography (CT) were performed in 46 patients. MRI, MRA, ENT and CT showed normal findings in the majority of the AH-patients (*n* = 38/46), but a small patient group (*n* = 8/46) showed inflammation and thickening of the mucosal wall in the sinuses observed in MRI (*n* = 4/8), MRA (*n* = 1/8), and CT (*n* = 7/8).

### AH pathophysiology

The pathophysiology of AH is still unknown, but speculative hypotheses have been proposed. The most frequently discussed mechanism for AH is that the changes in the cabin pressure during take-off and landing may lead to sinus barotrauma, local inflammation, and thereby development of AH [2–7, 9–14, 18–21, 24–27, 32, 35, 39, 40]. Due to possible variations in the anatomical and structural construction in the individual ethmoid sinuses, including ethmoid cells, these patients cannot equalize the pressure during the take-off or landing [7]. Ethmoid cells are innervated by branches of the trigeminal nerve, where these nerve endings may trigger a stimulus as a consequence of sinus barotrauma and thereby inflammation due to the lack of pressure equalization [7]. This may lead to the characteristic of pain localized in the fronto-orbital region [7].

So far, there has been only one experimental study that has attempted to investigate the mechanism in AH [25]. Prostaglandin E_2_ (PGE_2_) has been shown to be significantly high in AH-patients during a simulated flight when compared with healthy subjects. It is speculated that PGE_2_ is elevated due to local inflammation, which may cause vasodilation in the cerebral arteries and induce AH [25]. Based on one case occurred during landing, reversible cerebral vasoconstriction syndrome (RCVS) has been advanced as a possible mechanism in the pathophysiology of AH [23].

Some flight passengers develop anxiety of flying, which may have a psychological impact on the passengers during flight travels [12, 25]. The stress hormone cortisol has also been shown to be significantly elevated in AH-patients during a simulated flight when compared to healthy subjects indicating a physiological response during an AH-attack [25]. In addition, hypoxia has also been considered to be one of the other factors that may affect the development of AH [2, 25, 26].

### AH treatment

#### Pharmacological treatment

According to the literature, 79 AH-patients have taken medications in order to relieve the headache pain [2, 4, 6, 9, 11, 12, 16, 17, 19, 21, 26, 28, 35]. The medications were naproxen (*n* = 24/79), triptans (*n* = 12/79), paracetamol (*n* = 11/79), dipyrone (*n* = 7/79), ibuprofen (*n* = 6/79), unspecified NSAIDs (*n* = 4/79), nasal decongestant (*n* = 4/79), aspirin (*n* = 3/79), antibiotics (*n* = 2/79), antihistamine (*n* = 2/79), oxymetazoline (*n* = 1/79), and loxoprofen (*n* = 1/79). Relieving effects of the medications were reported by naproxen (*n* = 24/24), triptans (*n* = 9/12), paracetamol (*n* = 1/11), ibuprofen (*n* = 3/6), nasal decongestant (*n* = 1/4), aspirin (*n* = 1/3), antibiotics (*n* = 1/2), antihistamine (*n* = 2/2), and oxymetazoline (*n* = 1/1) [2, 6, 9, 11, 16, 17, 19, 26, 28, 35].

#### Non-pharmacological treatment

A small group of AH-patients (*n* = 35) has used self-administered maneuvers such as pressure on the headache pain site (*n* = 19/35), Valsalva maneuver (*n* = 11/35), relaxation methods (*n* = 3/35), chewing (*n* = 1/35), and extension of the ear lobes (*n* = 1/35) [6]. The maneuvers had shown varying effects since only seven patients experienced a reduction in the pain intensity [6].

## Discussion

### Literature search

In this systematic review, 39 articles were included. The authors decided to include all types of articles as the literature within AH is very limited. Therefore, the main purpose was to present and discuss the current evidence on diagnosis, pathophysiology, and treatment of AH. However, a restriction was made in the literature search strategy where only English articles were included. Articles that have not been indexed in PubMed, Scopus and Embase, may have not been included in this review. Despite of this, our systematic review presents 275 AH-patients, which, together give a stereotypical clinical picture of AH.

### AH diagnosis

The cumulative analysis in this review revealed that 127 females (46%) and 148 males (54%) have suffered from AH (275 patients in total). At present, it is difficult to conclude whether gender affects the development of AH. By instance, there was a predominance of males in few studies [4, 6, 26], whereas there was a predominance of females in the study by Bui et al. [2]. Gender difference in some primary headaches has been documented [41, 42], and if similar difference is identified in AH, it will provide knowledge of which particular gender is more susceptible to AH. The mean diagnosis age for AH was 28.7 ± 4.8 years (*n* = 275/275), while the age at first AH-attack was 26.4 ± 3.8 years (reported by 163/275 patients). These data may indicate that the diagnosis age of AH and the first AH-attack may occur in a relatively young age regardless of gender. There is no explanation on association of age with AH. This needs further investigation to identify whether it is related to pathophysiology of AH or demographic characteristics of travelers.

The stereotypical clinical symptom of AH is an intense unilateral pain locating in the fronto-orbital region. The pain is very severe and is often described as 8–10 on a pain scale from 0 to 10, where 0 is no pain and 10 is the worst imaginable pain [2, 4, 6, 9, 25, 35, 40]. The pain disappears within 30 min in most cases, where it seems that the AH duration might correspond to the duration of take-off and landing. However, there is a smaller group of AH passengers who experience a second-phase headache, which is a mild headache that may last hours to days; but, it may not be considered as a direct continuation of the intense headache that occurs within the airplane.

Onset of AH was found mostly during the descending phase in 210 patients (*n* = 210/275), followed by ascending phase in 33 patients (*n* = 33/275) [2, 4, 6, 7, 9, 11, 15–17, 19, 21, 25–28, 35–37, 39, 40], and only 18 patients (*n* = 18/275) were found to report it both during descending and ascending phase [2, 4, 5, 7, 11, 13, 26]. Based on the findings, AH was found to occur in 138 patients (*n* = 138/275) [5, 15, 17, 19, 28, 35–37] since their first flight experience.

AH is experienced without accompanying symptoms in almost all cases; but, there are few cases that cannot be considered as part of the stereotypical clinical symptoms of AH [13, 18]. In the current literature, there are 54 patients suffering from migraine [2, 4, 6, 7, 18]. Although there are many who suffer from migraine, it does not indicate that there is a direct link between migraine and AH [2]. In addition, these migraine patients have reported that they experienced AH and not migraine attacks (2,4,6,7,18). Furthermore, 22 patients suffering from tension type headache, did also report a pure AH-attack and not a tension type headache-attack [6, 12, 21]. In relation to sinus infections, only few cases have been reported, where there was an active sinus infection in the passengers who experienced AH [16]. This indicates that AH is a separate headache that is not associated with other headaches and conditions; but, it is currently unclear whether certain headaches or conditions (such as stress and anxiety) are potential risk factors for developing AH.

In 2013, AH was formally classified by IHS, where diagnostic criteria for AH were established [1]. This makes it possible for future studies to use the diagnostic criteria for harmonizing the research within this field (e.g. Bui et al. [2, 25]). It will also make it easier for doctors to diagnose AH at clinic as the criteria can be used identically.

### AH pathophysiology

Berilgen et al. [7] were the first to suggest “sinus barotrauma” as a potential mechanism underlying AH, which has been a key element in the subsequent discussion for AH mechanisms [2–5, 9–14, 18–21, 24–27, 32, 35, 39, 40]. It is well known that imbalance between atmospheric pressure changes and pressure inside the sinuses can cause tissue damage [43–45]. This means, for example, that the pressure in the sinuses is lower than the cabin pressure during the landing [6, 26]. At take-off, the cabin pressure is lower than the sinuses [6, 26]. It has also been shown that the cabin pressure changes during a flight travel, where the cabin pressure will decrease with around by 8 hPa for every 300 m the airplane increases in altitude [46]. The normal altitude for airplanes is 2500 m with a stabilized cabin pressure at 846 hPa [46].

Berilgen et al. [7] suggest that the first-degree sinus barotrauma is the key mechanism underlying AH. During the first-degree sinus barotrauma, nerve endings in the ethmoid sinuses, which are invaded by the trigeminal nerve [47], are affected. This causes pain in the fronto-orbital region [7, 48], which explains that the pain is mainly experienced in the orbital region [2, 4–13, 15–18, 21, 25–28, 35]. The proposal of first-degree sinus barotrauma may be reasonable as it is explained by the fact that neurological examinations, such as CT, MRI, MRA and ENT, have shown normal sinus conditions in several studies [5, 7–13, 15, 17, 18, 21, 26, 35]. However, few studies have shown cases of thickened nasal mucosa among 2 AH-patients with allergy and chronic rhinosinusitis [16, 19]. The patient with allergy was treated with antihistamines and subsequently experienced headache-free flight travels [16]. Data from these studies indicate that thickened nasal mucosa resulted from the allergy and chronic rhinosinusitis and not as a result of the flight travels. Coutinho et al. [17] suggest that MRI should be used to rule out other conditions including sinus barotrauma. This indicates that Coutinho et al. [17] have not taken into account the degrees of sinus barotrauma as those are only the second-degree and third-degree that show thickened nasal mucosa [45]. Relative to the pain of AH, the pain usually disappears within 30 min [2, 5–13, 15–19, 21, 25–27, 35], which is consistent with the short-term pain at the first-degree sinus barotraume [45]. However, neurological examinations were not performed on all the presented 275 AH-patients in this review and this should be considered in the future for proper diagnosis of AH at clinic.

It is still unknown which specific substances play a role in the mechanism of AH. Bui et al. have speculated that vasodilation may occur in the cerebral arteries during an AH-attack [25]. One of the substances investigated in that study was PGE_2_, where PGE_2_ levels were significantly higher in a group of AH-patients compared with healthy subjects during a simulated flight in a pressure chamber [25]. Wienecke et al. have shown that PGE_2_ can induce vasodilation in the cerebral arteries and headache in healthy subjects by infusion of PGE_2_ [49]. Authors suggested that PGE_2_-induced headache might be due to activation and sensitization of cranial perivascular sensory afferents [49]. As local inflammation may occur in the sinuses during an AH-attack [2–7, 9–14, 18–21, 24–27, 32, 35, 39, 40], it may be reasonable to consider that PGE_2_ increases as a consequence of this inflammatory condition [25]. This makes PGE_2_ an interesting potential biomarker for AH and infusion of PGE_2_ in AH-patients in future studies may provide knowledge whether PGE_2_ play a role in the AH mechanism.

The theoretical mechanism of AH is mainly based on barotrauma [2–7, 9–14, 18–21, 24–27, 32, 35, 39, 40] and possibly vasodilation in the cerebral arteries [25]. A study by Hiraga et al. [23] considers the opposite. The authors point out that vasoconstriction may be the cause of AH. The female patient in their study experienced a headache, which lasted for several hours and days when they performed a neurological examination and contraction of her cerebral arteries was seen. Mainardi et al. [22], however, proposed that patient’s symptoms do not match with the diagnostic criteria for AH and hence it is unclear whether vasoconstriction in the cerebral arteries can be a cause or a consequence of AH. For future studies, it would be valuable to use imaging techniques during a real or simulated flight travel to investigate whether vasoconstriction or vasodilation occurs in the cerebral arteries during an AH-attack.

It has been shown that cortisol levels were significantly higher before and during a simulated flight in a small group of AH-patients compared with a healthy matched group tested in a pressure chamber [25]. Some AH-patients reported that they felt stressed and had anxiety during the simulated flight [25]. In addition, Kararizou et al. [12] has also presented an AH-patient with anxiety. This might indicate that psychological aspects can also contribute in AH [12, 25]. However, flight passengers frequently present stress and anxiety in dealing with the flight and in most of cases they do not complain of AH. On the contrary, patients who do not have any negative emotional impact may start to present anxiety and stress when they experience AH. Therefore, stress and anxiety could be considered as a consequence of the fearing to develop AH instead of being one of principal pathogenetic mechanisms of AH. If anxiety and stress did have a predominant role in the pathogenesis of AH, then it should be expected that AH may occur in almost every flight, which is not the case.

It may be difficult to run experiments during a real-time flight travel both from practical points and also safety issues. However, an experimental model might be a reasonable alternative that can be used to induce AH, which allows investigating more physiological aspects of AH. Bui et al. [25] used a pressure chamber to study AH under controlled experimental conditions. In the chamber, pressure changes, which correspond to the changes during take-off and landing, are applied. This study presented occurrence of AH during simulated flight in those who suffer from this condition but not healthy controls [25]. The clinical symptoms of the simulated AH were found similar to the symptoms of the real-time AH-attacks [25]. This indicated that it is possible to use the pressure chamber as a platform experimental model to induce AH and to investigate AH under fully controlled conditions. Furthermore, it can also be used to investigate whether AH is associated with risk factors or comorbidities in future studies.

### AH treatment

There is no specific treatment plan for AH, that might be due to the fact that this headache is considered short lasting and terminated after the flight travel is over. However, evidences in the literature demonstrate that some passengers suffering from AH, have been required to take medications to subside the headache. This systematic review, only focused on pharmacological treatment, although there have been cases of non-pharmacological treatments [6].

Several flight passengers have been self-medicated in order to relieve AH, where only ibuprofen, naproxen, and triptans have been found the most effective [2, 6, 9, 11, 16, 17, 26, 35]. Only Berilgen et al. [26] have prescribed naproxen to their 21 subjects and all subjects experienced no headache after intake of naproxen before the start of their flights. Today, the effects of ibuprofen, naproxen, and triptans are based on the patient’s own narratives [2, 6, 9, 11, 16, 17, 26, 35]. Therefore, future studies seem necessary to provide a clear guideline as whether or not medications are actually needed and which patient will get the best benefit. Since most studies are based on case series and case reports, it is reasonable to perform future randomized controlled trials (RCTs) on pharmacological treatments of AH. Setting up a double blind RCT, where a control group is given placebo, and the other group, medication (ibuprofen, naproxen or triptans), can reveal the actual effect of medications on AH.

Based on the finding of this review, 6 flight passengers have taken ibuprofen, where 4 of those have experienced a relieving effect [6, 9, 16]. For the naproxen, 24 flight passengers have taken this medication with all experiencing a relieving effect [17, 26, 35]. If PGE_2_ has a central role in the mechanism of AH, then ibuprofen and naproxen seem reasonable choices, as these medications inhibit COX, reduce elevated levels of PGE_2_ during an AH-attack, and can abort headaches [25, 50].

Ipekdal et al. [11] performed a study in 2011 and included 5 patients who took triptans 30 min before flight travels and this prevented development of AH completely [11]. Cumulative findings of this systematic review showed that 12 patients have taken triptans in order to relieve AH [2, 4, 11]. Triptans are a class of drugs developed for abortion of migraine headaches [11, 51–53]. In relation to AH, Ipekdal et al. [11] have suggested that triptans may cause vasospasms during an AH-attack and thereby prevent it. There is, however, no evidence to present a mechanism-based explanation for use of triptans for AH.

Calcitonin gene-related peptide (CGRP) and vasoactive intestinal peptide (VIP) are believed to play a role in vasodilation in the cerebral arteries leading to development of migraine attacks [54]. Bellamy et al. have shown that CGRP and VIP levels are significantly reduced when patients take triptans during a migraine attack [54], which is associated with a significant relief of headache [54]. Future studies are required to investigate whether CGRP and VIP also play a role in development of AH. If AH is a result of vasodilation in the cerebral arteries, elevated levels of CGRP and VIP might be seen in AH and triptans that have shown some benefits for AH may act on these headache biomarkers.

The frequencies of AH-attacks are relatively high; 42 flight passengers experience AH in every flight travels and 39 flight passengers experience AH in more than 50% of the flight travels [4–6, 12, 19, 21, 27, 39, 40]. For the rest of 194 AH-patients presented in this systematic review it is not known how often AH occurs. Clinical trials, such as RCTs, seem necessary for better understanding of AH and the value of considering strategic plans for treatment or prevention of AH.

## Conclusions

Based on this systematic review, it is now evident that further studies are required to investigate AH systematically. Investigations may clarify unknown aspects of AH diagnosis that can be taken in updates of AH classification by IHS. Future experimental studies are also essential to further investigate proposed mechanisms underlying AH; barotrauma and vasodilation in the cerebral arteries, and also to investigate the biological effects of most used medications, ibuprofen, naproxen and triptans for alleviating of AH-attacks. These studies would advance our understanding of AH pathogenesis and value of treatment options that are not yet established. This would subsequently help millions of passengers suffering from this condition.
